# Recent Updates on Marine Cancer-Preventive Compounds

**DOI:** 10.3390/md19100558

**Published:** 2021-09-29

**Authors:** Sergey A. Dyshlovoy

**Affiliations:** Laboratory of Pharmacology, A.V. Zhirmunsky National Scientific Center of Marine Biology, Far Eastern Branch, Russian Academy of Sciences, 690041 Vladivostok, Russia; dyshlovoy@gmail.com

**Keywords:** marine natural products, cancer-prevention, polyphenols, alkaloids, carotenoids, lipids, macrolides, terpenoids, peptides, polysaccharides

## Abstract

The natural compounds derived from marine organisms often exhibit unique chemical structures and potent biological activities. Cancer-preventive activity is one of the rather new activities that has emerged and been extensively studied over the last decades. This review summarizes the recent updates on the marine chemopreventive compounds covering the relevant literature published in 2013–2021 and following the previous comprehensive review by Stonik and Fedorov (*Marine Drugs* **2014**, *12*, 636–671). In the current article, only the molecules having an effect on malignant transformation (or related pathway and molecules), cancer stem cells, or carcinogen-induced in vivo tumor development were considered to be “true” cancer-preventive compounds and were, therefore, reviewed. Additionally, particular attention has been given to the molecular mechanisms of chemoprevention, executed by the reported marine compounds.

## 1. Introduction

Cancer is a complex genetic disease, which appears as the uncontrolled growth of cells, first resulting in tumor formation and further gaining an ability to invade other tissues and organs (i.e., to metastasize) [1]. This pathological condition is stipulated by the alterations in gene structure and expression caused by various exogenous and endogenous factors. According to the most recent WHO report, cancer takes around 10 million lives annually [2]. In recent decades, tremendous progress in the diagnosis and treatment of different types of neoplasms has been achieved. Despite this, cancer is still one of the leading causes of death, worldwide, and especially in developed countries [3]. One of the reasons is the mutational background of this disease, which results in a huge heterogeneity in genotype and phenotype of tumor cells within the same cancer type, and even within the same tumor, in a single patient [4]. Therefore, cancer treatment is a challenging process that requires the use of multiple expensive medications and techniques. Additionally, anticancer therapy very often requires the application of cytotoxic targeted or untargeted chemotherapeutics. These drugs are often toxic to normal non-cancer cells and/or exhibit other side effects unrelated to their cytotoxicity [5]. Therefore, the prevention of cancer, rather than its treatment, is a highly favorable scenario for both patients and health care systems.

The marine environment is characterized by special conditions, such as lack of sunlight and oxygen, high pressure and salinity, low stable temperatures, etc. [6,7]. This determines the specific biochemistry of sea inhabitants that frequently results in the production of unique metabolites by marine micro- and macro-organisms. These molecules often exhibit promising biological activity, and some of them can be used to prevent and cure human diseases [8,9] as well as food additives and cosmetic ingredients [10]. Thus, today there are 16 drugs, approved for in-patient use, that were created and developed from natural marine-derived molecules. A significant proportion of these drugs—11 out of 16—is used in the treatment of different types of human cancers [11,12]. Furthermore, there is an impressive variety of food supplements developed from the extracts and semi-purified fraction of edible marine organisms, which were reported to be beneficial for human health [13,14]. Many of them were assumed to have antioxidant, immunostimulatory, and cancer-preventive properties [13,14,15,16,17,18].

Apart from the marine-derived drugs approved by the European and US authorities, there are many reports that clearly indicate the correlation of dietary habits and cancer risk [19]. Thus, a higher consumption of seafood was associated with lower risk for different cancers, especially colorectal and gastric cancer [20,21,22,23,24]. Interestingly, certain marine organisms are widely used in the traditional folk medicine of many Asian countries for the treatment of different pathological conditions, including cancer [25,26]. Some of the bioactive molecules responsible for the above-described health-beneficial effects have been isolated and characterized; others are still awaiting investigation. 

This review is based on a previous comprehensive review of the marine-derived small molecules exhibiting cancer-preventive properties, published in 2014, in *Marine Drugs,* by Stonik and Fedorov [27]. In that study, the authors analyzed papers from 2003–2013 that reported molecules exhibiting chemopreventive activity, in vitro or in vivo [27]. The current review covers the relevant literature published between June 2013 an September 2021 and gives the most recent updates on cancer-preventive marine compounds. Here, only the compounds capable of inhibiting malignant transformation in vitro or in vivo, and/or tumor formation in vivo, were assumed as cancer-preventive agents. Molecules for which only cytotoxic activity towards cancer cells has been reported were not considered cancer-preventive agents, but rather as chemotherapeutic compounds. Additionally, particular attention is given to their mechanisms of chemopreventive activity.

## 2. Carcinogenesis and Its Prevention

Data collected in recent decades clearly indicates cancer to be a very heterogeneous disease [4]. Every cancer type is genetically and biochemically different from each other, and therefore requires a specific therapeutic approach, or even better, a personalized treatment for every single patient [28,29]. The same is true for carcinogenesis. Thus, diverse alterations in different genes are involved in the genesis of various cancer types [30]. Moreover, different mechanisms may lead to the same oncogenic alteration in the same cell. However, there are three general steps of carcinogenesis that have been determined and described for the majority of tumors [31]:

**Initiation:** At first, a carcinogenic factor, e.g., a chemical agent, induces an irreversible (by forming DNA adducts) or reversible (epigenetic) modification of DNA or histones [1]. This may further lead to the mutation of DNA during its replication, the epigenetic promotion of oncogenes, or inhibition of tumor-suppressor gene activity [32].

**Promotion:** The affected cell is further divided, in order to expand the initiated clone and, therefore, to produce a larger population of cells bearing the genetic mutation acquired in the previous step. These cells have a higher risk of further genetic alterations and of consequent malignant transformation [1]. In this step, exposure to a tumor promoter is required. Normally, tumor promoters (e.g., phorbol ethers or epidermal growth factor (EGF)) themselves are not mutagens or carcinogens [33]. However, there are so-called “complete carcinogens” (e.g., benzo[a]pyrene) which are capable of both tumor initiation and promotion [34,35].

**Progression:** In this step, the cells start to express the malignant phenotype, which is characterized by uncontrolled growth and genetic instability. Additionally, the cells are able to secrete proteases, which facilitates their ability to invade other tissues and organs, thereby forming metastases [36].

To prevent cancer means, therefore, to inhibit one of the above-described steps of carcinogenesis. However, the majority of chemopreventive agents acts at the initiation step, i.e., via a neutralization of the carcinogen (stimulus) or via an attenuation of its DNA-damaging action. Apart from the genetic background, which already includes already existing inherited gene mutations (germline mutations), the newly acquired mutations in the body cells (somatic mutations) may result from the exposure to three carcinogen types. This includes physical (UV, ionizing radiation [IR]); chemical (cigarette smoke, benzo[*a*]pyrene, asbestos, aflatoxin, some hormones and hormone-like molecules, and many others), and biological carcinogens (e.g., papillomaviruses, hepatitis B and C, *Helicobacter pylori*, etc.) [37]. Chemopreventive agents may suppress a carcinogen or its secondary effects via different mechanisms. These mechanisms include an inhibition of chronic inflammation, which was reported as a predisposition to several cancer types; scavenging of reactive oxygen species (ROS) and other free radicals, which result from UV, IR, or chemical exposure and leads to DNA damage; stimulation of the innate immunity in order to recognize and eliminate a malignant-transformed cells; inhibition of oncogenic nuclear factors such as NF-kB and AP-1; and many others [38,39,40].

Another aspect, which is especially important in the models utilizing chemical carcinogens, is an effect on the xenobiotics biotransformation system. Drug-metabolizing enzymes are classified into two main groups, namely, phase I and II enzymes [41]. In phase I, the xenobiotic to be metabolized undergoes oxidation, reduction, or hydrolysis, which results in more polar metabolites. Then, these molecules enter phase II to undergo consequential conjugation with the substrate; this results in a non-reactive polar compound, which could be easily excreted from the body [42,43]. The main detoxifying enzymes of phase I are the members of the cytochrome P450 (CYP) family, whereas phase II enzymes include glutathione-S-transferases, quinone-reductases, *N*-acetyltransferases, sulfotransferases, methyltransferases, and others. Both phase I and II enzymes are located in the endoplasmic reticulum (ER). While the xenobiotics biotransformation (detoxification) system is supposed to neutralize toxic and harmful molecules, some chemicals, during this process, may form highly reactive and therefore carcinogenic compounds, e.g., epoxides, *N*-oxides, etc. Particularly often, this happens during oxidation in phase I, resulting in genetoxic compounds, which, however, in most cases will be further neutralized in phase II. Therefore, compounds capable of phase I-enzyme inhibition and phase II-enzyme promotion are often consideredchemopreventive agents; and it has been experimentally proven for a number of molecules. At the same time, it should be noted that phase II enzymes do not exclusively play a protective role and sometimes may also activate a carcinogen [44]. Thus, the above-mentioned assumption should be carefully examined in each particular case [45].

## 3. Polyphenols

Several studies have reported a cancer-preventive effect of marine polyphenols. These compounds are often isolated from a brown algae and have exhibited an impressive spectra of biological activities, including antioxidant and anti-tumor properties [46,47,48]. Previously, it has been reported that an extract of a brown alga *Ecklonia stolonifera* may attenuate a DMBA-induced chromosomal aberration [49]. Later, an anti-photocarcinogenic effect of the brown algae polyphenols has been reported in UVB-induced skin carcinogenesis in SKH-1 mice and suggested to be associated with an inhibition of pro-inflammatory COX-2 [50]. 

**Figure 1:**
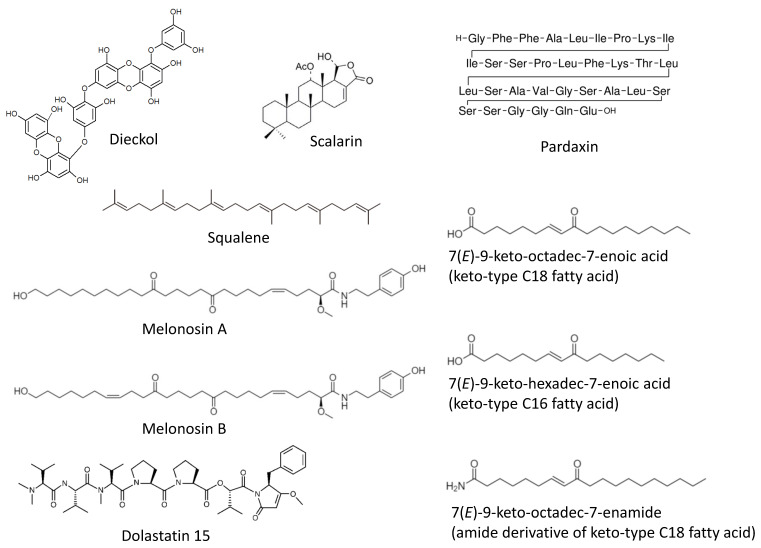
Marine cancer-preventive compounds: polyphenols, terpenoids, lipids and peptides.

Recently, a comprehensive in vivo-based study by Xiao et al. has reported chemopreventive properties of the phlorotannin **dieckol** (Figure 1) [51]. This marine polyphenol was isolated from a brown alga *Ecklonia cava* (belonging to Lessoniaceae family) and has been tested in a DMBA-induced skin cancer model in vivo. Thus, a daily dieckol treatment could significantly reduce a DMBA-induced tumor incidence, tumor volume, as well as tumor burden. The mechanism of action has been defined as (i) an inhibition of IkB/NF-kB signaling, (ii) reduction of inflammatory processes (i.e., suppression of IL-6, IL-1β and TNF-α), and (iii) antioxidant activity. The later was exerted via the induction of antioxidant enzymes (SOD, CAT, GPx) as well as increase of the glutathione level and a simultaneous inhibition of cytochromes p450 and b5 (phase-I detoxification enzymes). Additionally, an induction of pro-apoptotic (p53, Bax, caspase-3 and -9) and inhibition of anti-apoptotic (Bcl-2, COX-2, and TGF-β1) genes at transcriptional level has been detected in the drug-treated cells, suggesting dieckol promotes tumor cells apoptosis [51]. Of note, the antioxidant activity has been recently reported to be a major mechanism of the hepatoprotective activity of dieckol and related compounds in several liver damage models [52,53]. Thus, the hepatoprotective activity of dieckol has been reported in a tetrachloromethane-induced in vivo model. It was suggested to be exerted via an activation of antioxidant enzymes as well as via the anti-apoptotic activity of dieckol in hepatocytes [54,55]. One can suggest that the above-mentioned effect may also result in cancer-preventive properties of dieckol and other marine polyphenols in the chemical agents-induced liver injury models. Indeed, the study by Sadeeshkumar et al. published in 2017 has reported an in vivo chemopreventive activity of dieckol in rats in the model of N-nitrosodiethylamine-induced hepatocarcinoma [56]. The authors have shown dieckol to suppress the carcinogen-activated phase I enzymes (cytochrome P450, CYB5A, CYB5R, and CYPOR) and simultaneously increase an activity of phase II enzymes (GST and QR) therefore promoting a N-nitrosodiethylamine detoxification [56]. This marine polyphenol induces an apoptosis of malignant transformed cells (executed via intrinsic pathway) and inhibits MMP-2/9 and VEGF expression at both, mRNA and protein levels. Additionally, an inhibition of pro-inflammatory factors NF-kB and COX-2 has been detected. Based on these results the authors have speculated that dieckol may suppress angiogenesis, cancer cells invasion, and the carcinogen-induced inflammatory processes; however, in the current research these effects have not been further investigated [56].

## 4. Terpenoids

In 2020 Rajamani and colleagues reported a cancer-preventive effect of **squalene** (Figure 1) in the KBrO_3_-induced renal cell carcinoma model in vivo in rats [57]. Squalene is a triterpene isolated from brown algae and known to have potent medical properties [58,59]. The mechanism of its activity has been described as inhibition of multiple ROS-inducing factors as well as suppression of inflammatory, metastatic and survival processes. Thus, squalene could suppress HIF signaling in both VHL-wild type and VHL-mutant renal cell carcinoma, as well as inhibit a NF-kB-mediated inflammation. Additionally, it could decrease VEGF localization and a micronucleus frequency in polychromatic erythrocytes [57]. Even though no actual tumor formation has been detected in KBrO_3_-treated control rats, the above reported effects of squalene strongly suggest its chemopreventive activity in renal cell carcinoma executed via multiple mechanisms.

The receptor of advanced glycation end products (RAGE) is known to promote stress and survival as well as inflammatory pathways. This can lead to carcinogenesis, therapy resistance, proliferation and metastatic potential of pancreatic cancer cells [60]. Therefore, RAGE inhibitors have chemopreventive potential. Guzman et al. have shown that **scalarin** (Figure 1), a bioactive metabolite of the marine sponge *Euryspongia* cf. rosea, is able to inhibit a RAGE activity in pancreatic cancer cells and therefore may have chamopreventive properties [61,62]. The upcoming experiments should clarify this in the near future.

## 5. Lipids

In 2018 the group of Makarieva has reported an isolation of two new marine lipids–namely, **melonoside B** and **melonosins A** and **B** (Figure 1) from the marine sponge *Melonanchora kobjakovae* [63]. These molecules have been characterized as unusual lipids containing ω-hydroxy fatty acid amides. The authors have reported melonosins A and B to be capable of inhibition of oncogenic nuclear transcriptional factors AP-1 or NF-kB in JB6 Cl41 cells at noncytotoxic concentrations [63]. The observed effects have occurred independently from the p53 activity. Notably, in the model of JB6 Cl41 cells the activity of AP-1 and NF-kB has been previously reported to be critical for the EGF-promoted neoplastic transformation [64]. Hence, inhibitors of these nuclear factors may be considered potential cancer-preventive agents [64]. However, in the current study no further confirmatory experiments have been performed [63].

The Luesch et al. studied compounds capable of activating the cytoprotective Nrf2–ARE pathway [65]. This pathway, which is involved in the neutralization of ROS, is executed via induction of the antioxidant-response element (ARE). ARE controls and modulates a number of phase II detoxification enzymes [66]. Therefore, the small molecules capable of ARE-activation can be considered promising cancer-preventive agents. Using the ARE activity-guided fractionation, the authors have reported an isolation of three new monounsaturated fatty acids (MUFAs) from an edible green alga, *Ulva lactuca* [65]. Namely, an isolation of **keto-type C18 fatty acid**, **keto-type C16 fatty acid**, and the **amide derivative of keto-type C18 fatty acid** (Figure 1) has been reported. The keto-type C18 fatty acid could increase the expression of cytoprotective genes regulated by ARE (i.e., NADPH:quinone oxidoreductase 1, heme oxygenase 1, thioredoxin reductase 1, glutamate–cysteine ligase subunits, and the cystine/glutamate exchange transporter). The ARE-activation effect of the compound and the consequent induction of the ARE-controlled gene expression has also been observed, in vivo, in mice models [66]. The authors speculate that this is due to the ability of the isolated keto-type C18 fatty acid to stabilize Nrf2 via inhibition of its Keap1-mediated ubiquitination, which therefore leads to Nrf2 accumulation and translocation to the nucleus [65]. 

The Li et al. reported the chemopreventive effect of **n-3 polyunsaturated (omega-3) fatty acid** (Figure 1) (fish oil) diet in an DMBA-induced mammary gland cancer murine model, in vivo [67]. Thus, it was shown that a n-3 polyunsaturated fatty acid-rich diet during pregnancy reduces the risk of breast cancer in the offspring. This effect is related to the reduction of 17β-estradiol (E2) in both pregnant animals as well as female offspring. Additionally, the mechanism of action has been identified as the promotion of the p53 pathway and the inhibition of oncogenic NF-kB and JAK-STAT signaling [67]. The main compounds suggested to be responsible for the above-described activity are eicosapentaenoic acid (EPA) and docosahexaenoic acid (DHA). 

## 6. Peptides

**Pardaxin** (Figure 1) is a marine-derived antimicrobial polypeptide (H-GFFALIPKIISSPLFKTLLSAVGSALSSSGGQE-OH), which was isolated from the oceanic fish *Pardachirus marmoratus* (reviewed in [68]). Han et al. have studied a chemopreventive effect of this molecule in oral squamous cell carcinoma (OSCC) models [69]. Using a hamster buccal pouch model, they have shown that pardaxin administration can inhibit DMBA-induced carcinogenesis in vivo [69]. This effect is partially explained by the detected dose-dependent inhibition of prostaglandin E2 (PGE2) level, which are elevated in DMBA-treated animals and, therefore, by the inhibition of pro-inflammatory COX-2/PGE2 signaling. Additionally, the authors have shown that pardaxin induces a G2/M-phase cell-cycle arrest and the apoptosis of OSCC cells in vitro, which may also contribute to the cancer-preventive activity of this marine peptide [69].

The Sanyal et al. have studied the molecular mechanism of the chemopreventive action of the mollusk linear peptide **dolastatin 15** (Figure 1) isolated from the sea hare *Dolabella auricularia* (but actually produced by cyanophytes that are part of the mollusk’s diet), in combination with the COX-2 inhibitor celecoxib [70]. Previously, the same group has reported, in vivo, the cancer-preventive effect of the same marine peptide in a 1,2-dimethylhydrazine (DMH)-induced rat model of colon carcinogenesis [71]. The authors explained the observed effect by Bcl-2’s active site-targeting accompanied by the down-regulation of its expression. Additionally, the up-regulation of pro-apoptotic Bax, Apaf-1, cytochrome C, caspases, p53, and p21 was reported to play a role in this effect [71]. In a more recent study, the authors have further investigated the chemopreventive mechanisms of the drug combination, using the same model, and have reported an inhibition of the PI3K/AKT pathway by this drug combination [70]. This effect was achieved due to the targeting of the ATP binding sites of both PI3K and AKT. Additionally, treatment with dolastatin 15 plus celecoxib resulted in the increase of GSK-3β, pro-apoptotic Bad, PTEN, and transcription factor Egr-1, the downregulation of cyclin D1, as well as increased intracellular calcium and oxidative stress. This ultimately led to mitochondrial membrane-potential drop-down and the apoptotic death of colon epithelial cells exposed to the carcinogen [70].

## 7. Carotenoids

**Figure 2:**
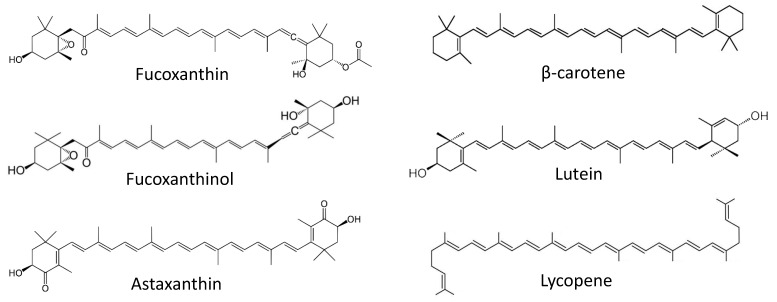
Marine cancer-preventive compounds: carotenoids.

**Fucoxanthin** (Figure 2) is a marine-derived carotenoid isolated from different brown alga species. Previously, this compound was reported to exhibit anticancer properties [72]. As many brown algae are consumed in food, the prevention of cancers of gastro-intestinal and colorectal system with fucoxanthin is of particular interest. In the recent years, a tremendous progress on the cancer-preventive activities of fucoxanthin and related compounds has been achieved by the Terasaki group. Recently, Terasaki et al. have reported the in-vivo cancer-preventive activity of this marine carotenoid in a colorectal carcinoma model [73]. The authors have speculated that an alteration of the gut microbiome plays a critical role in chemopreventive effect of fucoxanthin. In this model, an inflammation-associated carcinogenesis was initiated in the mice treated with azoxymethane/dextran sodium sulfate (AOM/DSS). Hence, in animals exposed to a carcinogen, treatment with fucoxanthin could reduce colorectal adenocarcinoma multiplicity. In line with this, the number of apoptosis-prone caspase-positive cells was increased in both colonic adenocarcinoma and mucosal crypts [73]. Interestingly, it has been noted that treatment with fucoxanthin led to the inhibition of *Bacteroidlales spp*. and *Rikenellaceae spp*. and, at the same time, promoted *Lachnospiraceae spp*. in gut microbiota. Remarkably, the authors have shown that administration of *Lachnospiraceae spp*. (achieved via gavaging, with suspension, the feces of mice receiving fucoxanthin) reduced a number of colorectal adenocarcinomas in carcinogen-exposed mice. Based on this, the conclusion of a critical role of the gut microbiome in the anticancer effect of fucoxanthin in inflammation-associated colorectal carcinoma has been made.

Earlier, in 2017, the same group reported the cancer-preventive effect of **fucoxanthinol** (Figure 2), which is an intestinal metabolite of fucoxanthin [74]. Notably, orally consumed fucoxanthin undergoes a deacetylation in the small intestine (under action of intestinal esterases), which results in fucoxanthinol. This has been observed in both mice and humans [74,75]. The authors have examined the effect of fucoxanthinol on colorectal cancer stem cells (CCSCs). Cancer stem cells (CSC) are tumorigenic cells that can persist in tumors and, due to their distinct properties, are often unaffected by chemotherapy. Therefore, CSC may later give rise to relapsed tumors and metastases [76]. Hence, compounds capable of killing CSC can be considered chemopreventive agents [77]. In this research, fucoxanthinol was capable of suppressing the growth of several CCSCs cell lines or CCSC-like cells in vitro and in vivo, presumably via the inactivation of Akt (inhibition of p-Akt), and the down-regulation of PPARβ/δ and PPARγ proteins. In ta CCSCs mice xenograft model, fucoxanthinol was able to slow down tumor progression. Therefore, the cancer-preventive properties of fucoxanthin administrated orally (e.g., via a brown alga-rich diet) in related models could, at least in part, be explained by the activity of its intestinal metabolite fucoxanthinol [74].

Very recently, in 2021, the Terasaki group characterized an effect of **fucoxanthinol** (Figure 2) on pancreatic ductal adenocarcinoma cancer cells, generated from a Syrian golden hamster exposed to N-nitrosobis(2-oxopropyl)amine [78]. The generated cell lines were characterized as papillary adenocarcinoma, well-differentiated tubular adenocarcinoma, moderately differentiated tubular adenocarcinoma, and poorly differentiated adenocarcinoma. Fucoxanthinol was able to effectively suppress the growth of all these cells. This effect was stipulated by the inhibition of cell cycles, chemokine and integrin levels, the suppression of actin polymerization, and the disruption of microtubule organization [78]. The treatment led to the inhibition of PI3K/AKT and TGF-β signaling pathways, and ultimately resulted in cell death via simultaneous apoptosis and anoikis. Thus, fucoxanthinol could eliminate the cells that underwent malignant transformation, which was considered a chemopreventive effect. 

In 2017 the same group reported **fucoxanthinol** (Figure 2) to induce anoikis (an anchorage-dependent apoptosis) in human colorectal cancer ecells in vitro th via inhibition of both integrin β1 signaling and focal adhesion kinase (FAK) [79,80]. More recently, in 2019 and 2020 Terasaki et al. have shown, in vivo, anoikis to be a mechanism of fucoxanthin cancer-preventive activity in a model of AOM/DSS-induced colon carcinogenesis [79]. Thus, a fucoxanthin-rich diet suppressed the number and size of polyps in the mice exposed to AOM/DSS as well as reduced the rate of colonic lesions and their multiplicity. The incidence and multiplicity of colonic adenocarcinoma was also reduced in mice receiving fucoxanthin. In line with these results, the suppression of such anoikis markers as integrin β1, p-FAK, p-paxillin, and the simultaneous caspase-3 activation was observed in the fucoxanthin-treated group. However, this effect was more pronounced in colonic adenocarcinoma cells in comparison with colonic mucosal crypts cells [79,80]. Thus, an induction of cancer epithelial cells anoikis was postulated as the main mechanism of fucoxanthin’s chemopreventive action in colon carcinogenesis models [80].

**Astaxanthin** (Figure 2) is another carotenoid found in various marine organisms [81]. Han et al. have reported its inhibitory effect on *Helicobacter pylori*-induced inflammation and oncogenesis in gastric mucosal tissues of mice [82]. The experiments were performed in in vivo models. *H. pylori* is a well-known risk factor of pancreatic cancer. Prolonged exposure of gastric epithelium cells to this bacterial infection results in long-term inflammation which may lead to carcinogenesis [83]. The astaxanthin-supplemented diet attenuates an *H. pylori* infection-induced activation of myeloperoxidase, expression of lipid peroxide, proinflammatory cytokine IFN-γ and c-myc oncogene as well as an expression of cyclin D1 [82]. Therefore, astaxanthin could protect gastric mucosal tissues from inflammatory and oncogenic responses as well as from the oxidative damage resulting from gastric *H. pylori* infection. Further studies should uncover the actual chemopreventive effect of astaxanthin and other marine carotenoids in the prevention of gastric cancer. 

Srinivasan and colleagues have reported the cancer-preventive effect of alyophilized carotenoids-rich *Dunaliella salina* extract, administrated as a food supplement, in a mammary cancer model in vivo [84]. In this research, female Wistar rats were treated with DMBA in order to induce mammary carcinoma. The treatment with the *Dunaliella salina* extract was able to decrease the levels of hormonal receptors relevant for the progression of breast cancer (such as ER, PR and HER2), inhibit cell proliferation, down-regulate the expression of pro-inflammatory COX-2 and induce apoptotic markers. The investigated extract was also shown to be able to revert the activities of antioxidant enzymes and phase-II detoxification enzymes (which were suppressed under the DMBA treatment), as well as phase-I detoxification enzyme (activated under the DMBA treatment) [84]. Ultimately, the treatment with lyophilized *D. salina* extract has resulted in reduction of tumor incidence rate and tumor volume in the carcinogen-exposed animals. HPLC analysis of the extract identified **β-carotene**, **lutein**, and **lycopene** (Figure 2) to be the main molecules presumably responsible for its chemopreventive properties [84]. The precise composition of the extract, however, has not been established.

## 8. Macrolides

**Figure 3:**
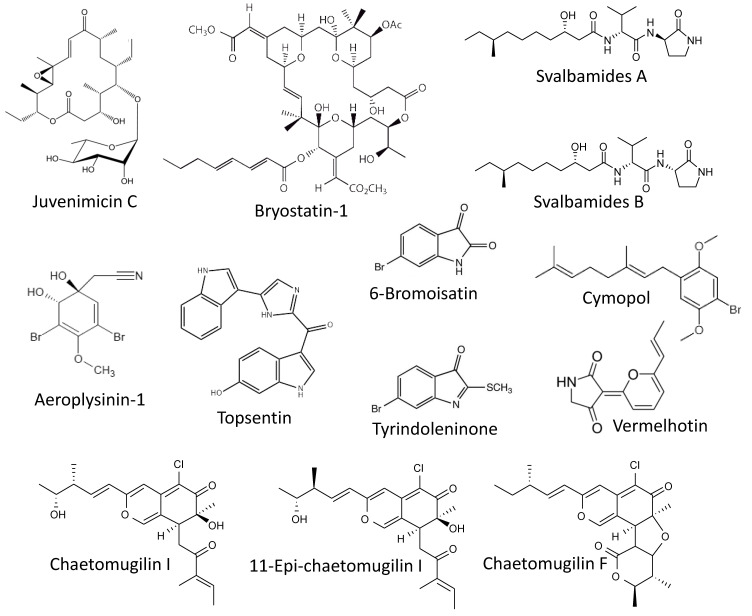
Marine cancer-preventive compounds: macrolides, alkaloids and other molecules.

Murphy et al. reported isolating of a new macrolide, **juvenimicin C** (Figure 3), from the marine bacterium *Micromonospora yangpuensis* [85]. Juvenimicin C was able to induce the phase-II enzyme quinone reductase 1 (QR1). This enzyme is responsible for two-electron detoxification and reduction of exogenous xenobiotics and carcinogens as well as endogenous ROS. Therefore, its activation is considered a marker of chemopreventive agents [86]. Additionally, juvenimicin C activated glutathione reductase, glutathione peroxidase and induced intracellular levels of glutathione. However, no further experiments confirming this suggested cancer-preventive activity have been performed.

Another well-known marine macrocyclic lactone, **bryostatin-1** (Figure 3), of bacterial origin and previously isolated from bryozoan *Bugula neritina* [87], has been examined for its chemopreventive properties by Salim and colleagues [88]. In this study, an association between chronic *Syphacia muris* parasite infection and 1,2-dimethylhydrazine (DMH)-induced colorectal carcinogenesis was been examined, in vivo, in a rat model. The authors have shown that treatment with bryosatin-1 reduced the formation of DMH-induced aberrant crypt foci. Additionally, histological examination has revealed a pronounced inhibition of the IgM, Ki67 and caspase-3 expression in the colorectal epithelium as well as serum IgM and IgG of the animals receiving bryosatin-1. The treatment has restored antioxidant properties of the cells. Finally, it has been shown that a DMH-treatment combined with a *S. muris* infection provoked an upregulation of pro-inflammatory COX-2 and simultaneously resulted in down-regulation of the tumor-suppression *APC* gene mRNAs in colorectal mucosa. Bryostatin-1 could reduce these above described negative effects (oxidative stress, inflammation, and antiapoptotic factors), which ultimately resulted in the reduction of the colorectal carcinogenesis [88].

## 9. Alkaloids

Another two potent small molecules capable of a QR1 enzyme induction are **svalbamides A** and **B** (Figure 3). These alkaloids were isolated by Du et al. from the sediment-derived arctic bacterium *Paenibacillus* sp. [89]. Svalbamides A and B could activate QR1 at their non-cytotoxic concentrations in the murine hepatoma model (Hepa1c1c7 cells), and therefore were suggested to be chemopreventive agents [89]. Though, no further validation experiment has been performed.

Park et al. have reported the cytotoxic activity of **aeroplysinin-1** (Figure 3) in colon cancer in vitro [90]. Aeroplysinin-1 is a brominated small molecule isolated from the marine sponge *Aplysina* sp. [91]. The authors described the anti-proliferative activity of aeroplysinin-1 in DLD-1 cells due to the attenuation of Wnt/β-catenin signaling exerted via promotion of β-catenin degradation [90]. It is known that the development of colorectal cancer is associated with mutation in the adenomatous polyposis coli (*APC*) gene. This mutation leads to accumulation of a nuclear β-catenin and ultimately results in the expression of a number of genes involved in colorectal tumorigenesis, tumor development and metastasis [92,93,94]. Thus, the degraders of β-catenin may have a potential cancer-preventive function. Indeed, aeroplysinin-1 treatment has promoted elimination of colorectal cancer cells, although no actual chemopreventive activity has been reported for this compound [90].

Esmaeelian and colleagues have studied the chemopreventive effect as well as the side effects of the brominated indoles **tyrindoleninone** and **6-bromoisatin** (Figure 3) in vivo [95]. These compounds were previously isolated from an Australian marine mollusk, *Dicathais orbita,* by the same group [96]. The effect was evaluated using the azoxymethane (AOM)-stimulated early-stage colon cancer model in C57BL/6 mice. In this model, daily oral administration of both tyrindoleninone and 6-bromoisatin as well as crude *Dicathais orbita* extract enhanced an apoptosis of the epithelial colon cells exposed to the carcinogen (AOM). Notably, in this study, the authors used a 6-h short-term treatment with AOM, thus no actual tumor formation could be observed in either group. Despite this, since AOM is an established genotoxic agent with carcinogenic properties [97], the authors have concluded that in a long-term exposure model, treatment with the isolated brominated indoles may result in an antitumorigenic effect [95]. Further validation studies confirming this speculation are warranted.

Hwang and colleagues have reported the photoprotective activity of the marine alkaloid **topsentin** (Figure 3) in human keratinocyte HaCaT cells [98]. Topsentin is a bis(indole)alkaloid isolated from the marine sponge *Spongosorites genitrix* [99]. It was shown that topsentin suppresses expression of UBV-induced pro-inflammatory COX-2 and prostaglandin E2 (PGE2), as well as the downregulation of AP-1 activity and MAPK signaling. Additionally, topsentin suppressed expression of the miRNA, miR-4485, and subsequently inhibited TNF-IP2 and TNF-α in UVB-irradiated keratinocytes. It has been previously postulated that inflammatory processes as well as the activation of MAPK and especially of AP-1 nuclear factor may contribute to the malignant transformation of epithelial skin cells [100,101,102]. Therefore, based on the generated results, the authors speculate that topsentin has a photoprotective effect and, consequently, may protect the skin from UVB-induced cancer. Further experiments are required to confirm this suggestion.

The Kittakoop group described an in vitro anti-inflammatory activity of the marine alkaloid vermelhotin [103]. This compounds has been previously isolated from the marine-derived fungi *Nodulisporium* sp. and CRI247-01 [104]. The authors have shown vermelhotin to inhibit NO production in LPS-stimulated macrophages. This effect has been exerted by the selective inhibition of p38 kinase, which resulted in inhibition of iNOS expression [103]. The generated data suggests vermelhotin to have an anti-inflammatory activity, which may also result in prevention of several cancer types as it has been described above. However, this speculation should be further confirmed experimentally.

## 10. Polysaccharides

Kokoulin et al. isolated and characterized a new **sulfated D-fucan** from the marine bacterium *Vadicella arenosi* KMM 9024^T^ [105]. The structure of polysaccharide was identified as a regular α-(1→3)-linked D-fucan sulfated at position O-2 of fucopyranosyl residues; it has been reported as the very first sulfated fucan found in bacteria [105]. The authors have reported the in vitro cancer-preventive activity of this molecule. Thus, the isolated polysaccharide could inhibit a malignant transformation of JB6 P^+^ Cl 41 murine cells. These cells are able to undergo the EGF-induced malignant transformation and consequently to form anchorage-independent colonies in soft agar; therefore, this well-established model is widely used for identification of cancer-preventive agents [106].

**Figure 4:**
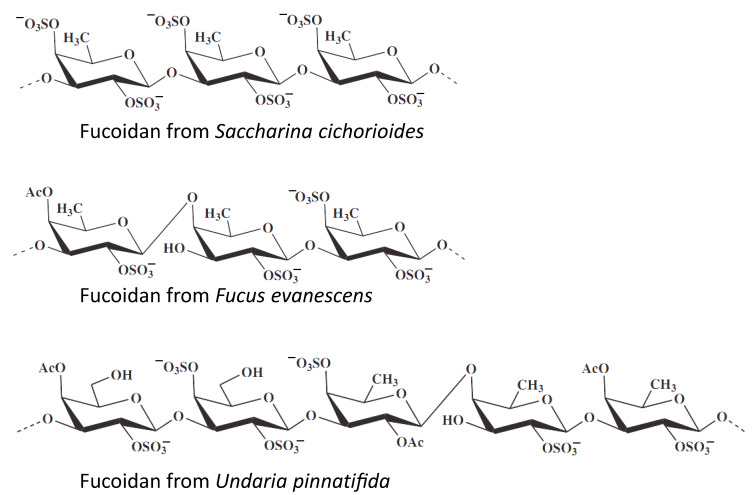
Marine cancer-preventive compounds: polysaccharides.

The same in vitro model of chemopreventive activity evaluation was utilized by Vishchuk at al., who studied te anticancer activities of **fucoidans** isolated from three brown algae, namely *Saccharina cichorioides*, *Undaria pinnatifida*, and *Fucus evanescens* (Figure 4) [107]. Fucoidans are sulfated polysaccharides found in brown algae and which exhibit a number of biological activities. In this study the authors have shown that highly sulfated (1→3)-α-L-fucan from *S. cichorioides* demonstrates the most pronounced cancer-preventive activity in the model of EGF-induced malignant transformation of JB6 P^+^ Cl41 cells [106]. Additionally, the authors have shown isolated fucoidans to have an inhibitory effect on anchorage-independent colony formation from the single cell, which have already undergone a malignant transformation. Such activity has been shown in human colon and breast cancer, as well as in melanoma models [107].

The Zhu et al. reported the cancer-preventive activity of te marine polysaccharide fucoidan, isolated from the brown alga *Fucus evanescens* [108]. This cancer-preventive activity has been shown in the above-mentioned JB6 P^+^ Cl41 cell model. The authors suggest TOPK kinase to be one of the direct targets of fucoidan, and have therefore speculated its chemopreventive activity to be exerted via an inhibition of TOPK/ERK/MSK1 signaling. In line with this, fucoidan was able to inhibit colon cancer tumor growth in a mice xerograph model, which was stipulated by the inhibition of TOPK activity in the tumor tissues [108]. 

A fraction of **sulfated polysaccharides** from *Ulva lactuca* has been studied for its chemopreventive activity in the hepatocarcinoma (HCC) rat model [109]. In this model, HCC was initiated in rodents by diethylnitrosamine and then further promoted by phenobarbital treatment. The authors have shown that the treatment with the sulfated polysaccharides fraction of *U. lactuca* reduces toxin-induced oxidative injury of the liver due to the modulation of several enzymatic and non-enzymatic (e.g., direct scavenging of ROS) hepatic antioxidant defense pathways. Additionally, this fraction inhibits abnormal proliferation of the cells and induction of apoptosis of the malignantly transformed and fast proliferating cells, therefore exhibiting a chemopreventive activity [109].

## 11. Other Molecules

The Chang group reported an isolation of the previously known compounds **11-epi-chaetomugilin I**, **chaetomugilin I**, and **chaetomugilin F** (Figure 3) from endophytic fungus *Chaetomium globosum* [110], which has been described as a symbiotic microorganism associated with several marine macroorganisms (reviewed in [111]). These molecules have been previously described by other groups [112,113,114]. The compounds could inhibit the (TNF-α)-induced NF-κB activity in HEK293 cells at their low micromolar non-cytotoxic concentrations. Based on this data, cancer-preventive properties of these compounds have been proposed, however, no further experiments confirming this activity have been performed [110]. 

Recently, Luesch et al. published a comprehensive study reporting the in vivo anti-inflammatory effect on **cymopol** (Figure 3) [115]. Cymopol, a brominated phenolic compound isolated from a marine green alga *Cymopolia barbata*, was able to increase the antioxidant status of the intestine cells via modulation of antioxidant response mediated via transcription factor Nrf2. This activatory effect was achieved via direct interaction of cymopol quinone, a bioactiveted cymopol metabolite, and cysteine residues Keap1, which is known to be a cytoplasmic repressor protein of Nrf2 factor. Furthermore, cymopol was able to inhibit inflammatory gene transcription in vitro in macrophages and fibroblasts, which appeared to be executed via an Nrf2-dependent pathway. In addition to the antioxidant effect, cymopol has shown anti-inflammatory properties in vivo in the DSS-induced inflammatory model. Finally, the investigation of mucosal-associated microbiome demonstrated some effects that may be beneficial for treatment of various diseases, thereby producing possible polypharmacological effects [115]. Since these antioxidant and anti-inflammatory effects have been of critical importance in the prevention of colorectal cancer, cymopol and related compounds have been suggested to have a chemopreventive activity. This speculation awaits further experimental validation.

## 12. Mixtures and Extracts 

The chemopreventive activities of the extracts of two edible algae—the **green alga *Capsosiphon fulvescens*** and the **brown alga *Hizikia fusiforme***—have recently been reported [116]. These algae are widely used as a food supplement as well as in traditional folk medicine in Asian countries. The Jeong group described cancer-preventive properties of these extracts in vivo in a model of azoxymethane-induced colorectal cancer in rats [116]. The diet supplemented with these extracts has resulted in reduction of aberrant crypt-foci formation as well as in decreased proliferating cell nuclear antigen labeling index in the colonic tissues. However, no chemical composition analysis of the extracts has been done. Due to the antioxidant properties of the extracts, the authors have suggested that the mechanism of their cancer-preventive activity can be a P450 2E1 (CYP2E1) pathway inhibition; however, no validation experiments have been performed [116].

Osuna-Ruiz et al. have studied the **extracts of green and brown algae *S. filamentosa****, **R. riparium*** and ***C. sertularioides*** collected from Sinaloa (Mexico) [117]. The authors speculate that these extracts exhibit cancer-preventive properties due to their antioxidant, antiproliferative and antimutagenic activities. Additionally, the authors have hypothesized that flavonoids and chlorophylls may be active compounds responsible for these reported chemopreventive properties. However, no further experiments confirming this suggestion were performed in this study. More recently, the same group investigated the composition of the studied extracts [118]. The authors speculate **polyunsaturated fatty acides**, **lutein**, **chlorophylls** as well as **β-sitosterol** to be the main components responsible for the previously reported chemopreventive properties of the extracts [118].

Burgos Hernández et al. suggest that the fractionated hexane and methanol extract of **octopus *Paraoctopus limaculatus*** contained molecules capable of a chemoprotective activity [119,120]. A chemical analysis of the extract revealed the presence of compounds containing double bonds as well as oxygenated molecules (alcohols, ketones, and ethers). The authors have made this speculation based on itsantimutagenic activity, which was detected in the bacterium-based assay with aflatoxin B1 as a mutagen. Additionally, an antiproliferative activity in murine B-cell lymphoma cells and an antioxidant activity in cell-free assay have been reported [119,120]. However, such speculationed chemoprotective properties require further experimental confirmation as no relevant assay in mammalian cells has been performed. 

## 13. Conclusions

Marine organisms have proven to be a very rich source of natural molecules possessing various biological activities. Among others, the cancer-preventive activity has attracted a lot of attention in recent decades. Over the last nine years, thirty four marine-derived compounds have been reported to exhibit chemopreventive properties. For some of these compounds the cancer-preventive activity in other models has been previously described. Overall, the absolute number on the molecules for which the above-mentioned activity has been reported for the very first time has reduced. At the same time, a significant number of the molecules for which the cancer-preventive properties have previously been described were further investigated over the past years. Furthermore, the main research priority has been shifted to compounds found in edible sources, for example, the properties of carotenoids and polysaccharides found in marine algae were extensively studied recently. Of note, the reported mechanisms of cancer-preventive activities were often related to their anti-inflammatory, anti-ROS or free-radical scavenging, anti-angiogenetic, and immunomodulatory activities. Additionally, several compounds have demonstrated an ability to inhibit the carcinogen-activating phase I enzymes (cytochromes p450) and simultaneously increase the activity of phase II enzymes, which was revealed to be beneficial for carcinogen detoxification. Finally, for several compounds, their apoptosis-inducing activity was suggested to be responsible for their cancer-preventive properties, which were executed via elimination of malignant transformed cells or cancer stem cells. Overall, marine natural compounds, and in particular those found in edible marine organisms, have much potential as chemopreventive agents, and could be consumed as part of a daily diet. Therefore, further studies of their activity and mechanisms of action utilizing various biological models should highlight the most potent and promising molecules for the development of new drugs and food additives.
